# Supplementary material for: Hot spot prediction in protein-protein interactions by an ensemble system
Source: BMC Syst Biol. 2018 Dec 31;12(Suppl 9):132. doi: 10.1186/s12918-018-0665-8 (PMC6311905; doi:10.1186/s12918-018-0665-8)
Supplement: Supplementary file 2 — All relevant MATLAB code. A simple Matlab implement of our predictor. (ZIP 19,742 kb) [file 12918_2018_665_MOESM2_ESM.zip › Additional file 2/Read ASA/ASA Property/SKEMPI/1JRHI.txt]

 NetSurfP - Protein Surface Accessibility and Secondary Structure Predictions

  

|  |  |  |
| --- | --- | --- |
|  |  | NetSurfP - Protein Surface Accessibility and Secondary Structure Predictions  Technical University of Denmark |

  


---

```
# For publication of results, please cite:
# A generic method for assignment of reliability scores applied to solvent accessibility predictions.
# Bent Petersen, Thomas Nordahl Petersen, Pernille Andersen, Morten Nielsen and Claus Lundegaard
# BMC Structural Biology 2009, 9:51 doi:10.1186/1472-6807-9-51
#
# Column 1: Class assignment - B for buried or E for Exposed - Threshold: 25% exposure, but not based on RSA
# Column 2: Amino acid
# Column 3: Sequence name
# Column 4: Amino acid number
# Column 5: Relative Surface Accessibility - RSA
# Column 6: Absolute Surface Accessibility
# Column 7: Z-fit score for RSA prediction
# Column 8: Probability for Alpha-Helix
# Column 9: Probability for Beta-strand
# Column 10: Probability for Coil
E S  1JRHI                  1    0.904 105.925  -0.236   0.003   0.003   0.994
E V  1JRHI                  2    0.327  50.275  -1.002   0.005   0.015   0.979
E P  1JRHI                  3    0.362  51.382  -0.805   0.005   0.015   0.979
E T  1JRHI                  4    0.540  74.953   1.100   0.018   0.019   0.964
B P  1JRHI                  5    0.179  25.414  -1.836   0.018   0.047   0.935
E T  1JRHI                  6    0.557  77.228   0.233   0.018   0.088   0.893
E N  1JRHI                  7    0.426  62.440  -0.143   0.005   0.262   0.733
B V  1JRHI                  8    0.074  11.389   0.340   0.003   0.718   0.279
E T  1JRHI                  9    0.293  40.639   1.070   0.001   0.900   0.099
B I  1JRHI                 10    0.055  10.194   0.981   0.001   0.959   0.040
E E  1JRHI                 11    0.262  45.859   1.348   0.001   0.959   0.040
B S  1JRHI                 12    0.030   3.563  -1.331   0.001   0.959   0.040
E Y  1JRHI                 13    0.283  60.541   0.307   0.002   0.816   0.182
E N  1JRHI                 14    0.276  40.377  -1.039   0.005   0.262   0.733
B M  1JRHI                 15    0.179  35.778  -0.108   0.004   0.138   0.858
E N  1JRHI                 16    0.335  49.117   0.150   0.004   0.616   0.381
B P  1JRHI                 17    0.133  18.816   0.520   0.001   0.900   0.099
B I  1JRHI                 18    0.074  13.690   0.801   0.001   0.959   0.040
B V  1JRHI                 19    0.028   4.257   0.676   0.001   0.959   0.040
B Y  1JRHI                 20    0.193  41.180   0.710   0.001   0.959   0.040
B W  1JRHI                 21    0.048  11.472  -0.199   0.002   0.816   0.182
E E  1JRHI                 22    0.389  67.923   0.709   0.004   0.420   0.576
B Y  1JRHI                 23    0.253  54.023   0.330   0.004   0.197   0.799
E Q  1JRHI                 24    0.564 100.784  -0.362   0.005   0.045   0.951
E I  1JRHI                 25    0.679 125.597  -2.169   0.005   0.015   0.979
E M  1JRHI                 26    0.432  86.503  -1.271   0.005   0.015   0.979
E P  1JRHI                 27    0.479  67.928  -1.542   0.005   0.015   0.979
E Q  1JRHI                 28    0.607 108.464  -1.388   0.005   0.045   0.951
E V  1JRHI                 29    0.477  73.253  -0.723   0.004   0.138   0.858
B P  1JRHI                 30    0.104  14.715   0.478   0.004   0.616   0.381
B V  1JRHI                 31    0.193  29.679   0.636   0.001   0.959   0.040
B F  1JRHI                 32    0.046   9.172   0.745   0.001   0.959   0.040
B T  1JRHI                 33    0.127  17.615   0.754   0.001   0.959   0.040
B V  1JRHI                 34    0.022   3.351   1.151   0.001   0.959   0.040
B E  1JRHI                 35    0.083  14.500   0.085   0.001   0.959   0.040
B V  1JRHI                 36    0.112  17.245   0.517   0.001   0.959   0.040
B K  1JRHI                 37    0.191  39.248   0.656   0.001   0.959   0.040
B N  1JRHI                 38    0.221  32.398   0.035   0.001   0.900   0.099
E Y  1JRHI                 39    0.289  61.674  -0.780   0.022   0.359   0.619
E G  1JRHI                 40    0.416  32.708  -1.093   0.020   0.205   0.775
E V  1JRHI                 41    0.467  71.732  -0.631   0.018   0.088   0.893
E K  1JRHI                 42    0.639 131.401  -0.684   0.018   0.088   0.893
E N  1JRHI                 43    0.610  89.231   0.403   0.018   0.088   0.893
B S  1JRHI                 44    0.221  25.854  -0.852   0.019   0.141   0.840
E E  1JRHI                 45    0.455  79.401   0.631   0.021   0.451   0.528
E W  1JRHI                 46    0.510 122.607   0.420   0.021   0.451   0.528
E I  1JRHI                 47    0.261  48.266   0.517   0.064   0.216   0.721
E D  1JRHI                 48    0.728 104.919   0.266   0.113   0.087   0.800
E A  1JRHI                 49    0.480  52.896   1.496   0.052   0.084   0.864
B C  1JRHI                 50    0.055   7.708  -0.988   0.064   0.216   0.721
E I  1JRHI                 51    0.443  82.048   0.590   0.069   0.386   0.545
E N  1JRHI                 52    0.483  70.653   0.023   0.021   0.279   0.699
B I  1JRHI                 53    0.175  32.467   0.437   0.020   0.205   0.775
E S  1JRHI                 54    0.552  64.694  -0.146   0.022   0.359   0.619
E H  1JRHI                 55    0.426  77.417   0.042   0.022   0.359   0.619
E H  1JRHI                 56    0.272  49.513   0.867   0.022   0.552   0.426
E Y  1JRHI                 57    0.443  94.690  -0.065   0.021   0.756   0.223
B C  1JRHI                 58    0.034   4.774  -0.001   0.021   0.756   0.223
E N  1JRHI                 59    0.333  48.751  -0.085   0.023   0.655   0.322
B I  1JRHI                 60    0.055  10.175   0.205   0.022   0.552   0.426
B S  1JRHI                 61    0.172  20.205   0.336   0.022   0.359   0.619
E D  1JRHI                 62    0.446  64.225  -0.438   0.021   0.279   0.699
E H  1JRHI                 63    0.497  90.459  -0.210   0.064   0.216   0.721
B V  1JRHI                 64    0.178  27.343  -0.869   0.021   0.279   0.699
E G  1JRHI                 65    0.401  31.567  -0.432   0.021   0.279   0.699
E D  1JRHI                 66    0.373  53.749  -0.660   0.004   0.138   0.858
E P  1JRHI                 67    0.315  44.741  -0.476   0.004   0.138   0.858
E S  1JRHI                 68    0.539  63.206  -1.490   0.005   0.262   0.733
B N  1JRHI                 69    0.222  32.574  -0.587   0.004   0.616   0.381
E S  1JRHI                 70    0.330  38.676   0.517   0.002   0.816   0.182
B L  1JRHI                 71    0.052   9.503   0.455   0.001   0.959   0.040
B W  1JRHI                 72    0.179  42.977   0.902   0.001   0.959   0.040
B V  1JRHI                 73    0.020   3.105   0.782   0.001   0.959   0.040
B R  1JRHI                 74    0.093  21.366   0.688   0.001   0.959   0.040
B V  1JRHI                 75    0.023   3.458   1.053   0.001   0.959   0.040
B K  1JRHI                 76    0.183  37.643   1.721   0.001   0.959   0.040
B A  1JRHI                 77    0.026   2.832   0.247   0.001   0.959   0.040
E R  1JRHI                 78    0.346  79.326   0.802   0.002   0.816   0.182
B V  1JRHI                 79    0.249  38.225  -0.288   0.004   0.514   0.481
E G  1JRHI                 80    0.557  43.867  -1.373   0.005   0.015   0.979
E Q  1JRHI                 81    0.729 130.217  -1.038   0.005   0.015   0.979
E K  1JRHI                 82    0.483  99.271   0.564   0.020   0.205   0.775
E E  1JRHI                 83    0.473  82.720   0.021   0.021   0.279   0.699
B S  1JRHI                 84    0.140  16.349  -0.723   0.021   0.279   0.699
E A  1JRHI                 85    0.492  54.240  -0.137   0.021   0.279   0.699
B Y  1JRHI                 86    0.237  50.540  -0.389   0.022   0.359   0.619
B A  1JRHI                 87    0.255  28.079  -1.104   0.022   0.552   0.426
E K  1JRHI                 88    0.459  94.334   0.075   0.022   0.552   0.426
E S  1JRHI                 89    0.338  39.578  -0.363   0.022   0.359   0.619
E E  1JRHI                 90    0.579 101.081   0.961   0.021   0.279   0.699
E E  1JRHI                 91    0.456  79.733   0.741   0.021   0.451   0.528
B F  1JRHI                 92    0.100  20.070  -0.949   0.023   0.655   0.322
E A  1JRHI                 93    0.404  44.488   0.345   0.023   0.655   0.322
B V  1JRHI                 94    0.250  38.486  -0.883   0.022   0.552   0.426
E S  1JRHI                 95    0.802  93.948  -0.056   0.005   0.015   0.979

Explain the output.  Go back.
```

---
